# Repurposing drugs for solid tumor treatment: focus on immune checkpoint inhibitors

**DOI:** 10.20892/j.issn.2095-3941.2023.0281

**Published:** 2023-11-06

**Authors:** Qingxu Liu, Long Li, Wan Qin, Tengfei Chao

**Affiliations:** Department of Oncology, Tongji Hospital of Tongji Medical College, Huazhong University of Science and Technology, Wuhan 430030, China

**Keywords:** Drug repurposing, immune checkpoint inhibitor, immunotherapy, tumor microenvironment

## Abstract

Cancer remains a significant global health challenge with limited treatment options beyond systemic therapies, such as chemotherapy, radiotherapy, and molecular targeted therapy. Immunotherapy has emerged as a promising therapeutic modality but the efficacy has plateaued, which therefore provides limited benefits to patients with cancer. Identification of more effective approaches to improve patient outcomes and extend survival are urgently needed. Drug repurposing has emerged as an attractive strategy for drug development and has recently garnered considerable interest. This review comprehensively analyses the efficacy of various repurposed drugs, such as transforming growth factor-beta (TGF-β) inhibitors, metformin, receptor activator of nuclear factor-κB ligand (RANKL) inhibitors, granulocyte macrophage colony-stimulating factor (GM-CSF), thymosin α1 (Tα1), aspirin, and bisphosphonate, in tumorigenesis with a specific focus on their impact on tumor immunology and immunotherapy. Additionally, we present a concise overview of the current preclinical and clinical studies investigating the potential therapeutic synergies achieved by combining these agents with immune checkpoint inhibitors.

## Introduction

Cancer remains a significant threat to human life and health. In recent decades immunotherapy has emerged as a potential “turning point” in tumor therapeutics that is facilitated by an increased understanding of tumor immune evasion, potentiation of the immune system, and reshaping of the immune microenvironment to prevent tumor immune escape^1^. Recently, several novel immunotherapeutic approaches have been developed, including immune checkpoint inhibitors (ICIs), oncolytic viruses, tumor vaccines, and chimeric antigen receptor T (CAR-T) cell therapy^2,3^. Among these novel immunotherapeutic approaches, ICIs have demonstrated clinical efficacy in enhancing immune system activity for the elimination of tumor cells, which led to the recognition of “cancer immunotherapy” as the 2013 Breakthrough of the Year by the editors of *Science*^4^. The emergence of immunotherapy has transformed the conventional approach and paradigm of cancer therapy. Indeed, immunotherapy has become the third revolution in tumor treatment after traditional chemotherapy and targeted therapy.

Owing to the complex nature of tumors and the diversity of individual immune environments, immunotherapy does not exhibit uniform therapeutic efficacy across all tumors and individuals; adverse effects may also vary^5,6^. The low responsiveness of ICIs in some patients with cancer can be attributed to several factors, including tumor heterogeneity, a lack of tumor-infiltrating T cells, an immunosuppressive tumor microenvironment (TME), lack of target antigen presentation, intrinsic T cell dysfunction, and cold tumor types. To address the current challenges in tumor immunotherapy, specific targets need to be identified, appropriate patients should be selected, and combination therapies must be applied^7^. The development of anti-tumor drugs is time-consuming and costly. Moreover, with the increasing number of drugs available in the market, pharmacoeconomic considerations are important in addition to efficacy and safety^8^. Drug repurposing, which involves the identification of new indications for existing approved drugs, provides a simplified research and development process^9^. A significant body of evidence suggests that a considerable number of drugs without current anti-tumor indications that are clinically safe and familiar to clinicians may possess anti-tumor effects^10,11^. Therefore, exploring novel pharmacologic effects of existing drugs is of interest among healthcare professionals to conserve medical resources, enhance patient outcomes, including prolonged survival and improved quality of life, and fully leverage the potential uses of conventional drugs. This review provides an extensive summary of advances in research involving the anti-tumor effects of non-tumor drugs in combination with immune checkpoint blockade therapy.

## TME phenotype and the impact on immunotherapy

The human immune system is responsible for immune surveillance. Specifically, the human immune system identifies and eliminates tumor cells that express tumor antigens on their surfaces. In some instances, however, tumor cells evade immune surveillance through various mechanisms. The TME is an intricate and heterogenous system comprised of various immune cells, biomolecules, and an extracellular matrix that has a significant role in immunotherapy^12^. The TME impacts the response to immunotherapy and is one of the critical drivers of tumor immune evasion. Immune cells present in the TME, such as natural killer (NK) cells, T cells, dendritic cells (DCs), macrophages, and myeloid-derived suppressor cells (MDSCs), interact with cytokines and chemokines to regulate tumor growth, invasion, and metastasis^13^. The cytokine spectrum in the TME influences T cell infiltration and impacts the outcome of tumor therapy. The TME is referred to as the seventh tumor marker and represents the vital battleground where the host immune system confronts cancer, and the two engage in dynamic interactions. Consequently, targeting immune cells in the TME to regulate tumor immunity has emerged as a crucial research focus^14,15^. The various immune evasion strategies used by cancer cells to evade detection and destruction include reducing immunogenicity by downregulating surface antigens, inhibiting T lymphocyte activity by upregulating immune checkpoints, recruiting immunosuppressive cells to the tumor immune microenvironment, and releasing metabolites to inhibit immune cell activity^16–19^. New immunotherapies, such as ICIs, tumor vaccines, cellular immunotherapy, and oncolytic viruses, have emerged as major factors in tumor immunotherapy^2,3^.

Recently, ICI therapy has achieved impressive results in the treatment of various malignancies^20^. Immune checkpoints tightly regulate immune system functioning^21^. Immune checkpoints either promote T cell activation and induce an immune response (stimulatory immune checkpoint molecules) or inhibit the immune response and prevent autoimmunity (inhibitory immune checkpoint molecules), thereby naturally regulating the human immune system. Tumor cells evade detection and immune system destruction by producing immune checkpoint molecules that hinder T cell activity, thus impeding their ability to eliminate cancerous cells^22,23^. Therefore, drugs targeting these inhibitory immune checkpoints enhance the immune system by blocking the inhibitory signals, ultimately leading to tumor elimination. To date, > 10 immune checkpoints have been discovered, with a focus on cytotoxic T lymphocyte-associated antigen-4 (CTLA-4) and the programmed death 1 (PD-1)/programmed cell death ligand 1(PD-L1) pathway^20^. In 2011 the FDA granted an initial approval for ipilimumab, an antibody drug targeting CTLA-4, making ipilimumab the first immunotherapy drug approved for the treatment of melanoma. In 2014 pembrolizumab was the first PD-1 inhibitor approved by the FDA for the treatment of melanoma and lung cancer. Atezolizumab was the first PD-L1 inhibitor approved for the treatment of bladder cancer in 2016^24,25^. Subsequently, an increasing number of immunotherapeutic drugs have gained marketing approval and research on tumor immunotherapy is ongoing.

Although immunotherapy has demonstrated significant anti-tumor effects, many malignancies evade immune surveillance through diverse mechanisms. In the context of immunotherapies, the terms “hot tumors” and “cold tumors” are used to describe the level of immune activity within a tumor. Hot tumors are characterized by a high level of immune activity within the TME. Hot tumors often have an inflamed TME, which is conducive to immune responses. This inflammation can be triggered by factors like a high mutational burden, viral infections, or other mechanisms that stimulate the immune system. Cold tumors, in contrast, have a low level of immune activity within the TME, which typically lacks the inflammation that exists in hot tumors. This finding may be due to factors, such as a low mutational burden, the absence of immune-stimulating signals, or the presence of immunosuppressive factors^26,27^. The TME has a critical role in determining the effectiveness of ICIs in cancer treatment. The TME is categorized into three primary phenotypes: immune-inflamed; immune-excluded; and immune-desert^7,28^. Each phenotype has distinct implications for the response to ICIs. An immune-inflamed TME is characterized by the presence of a robust and active immune response within the TME, as exists in melanoma and lung cancer. An immune-inflamed TME includes the infiltration of immune cells, such as T cells and antigen-presenting cells, into the tumor. An immune-excluded TME is characterized by the presence of immune cells at the periphery of the tumor, but these immune cells are unable to penetrate the tumor core, as exists in colorectal cancer. This finding can be attributed to physical barriers or immunosuppressive factors within the tumor. An immune-desert TME is characterized by the absence of significant immune cell infiltration into the tumor, as exists in pancreatic cancer and glioblastomas^29,30^. These tumors lack a detectable immune response within the TME. Understanding the TME phenotype is crucial for tailoring treatment strategies. Immune-inflamed tumors are most likely to respond to ICIs, immune-excluded tumors may require additional approaches to overcome barriers, and immune-desert tumors often necessitate innovative combination therapies to prime the immune response^30^. Therefore, the combination of immunotherapies with repurposed agents is increasingly viewed as a potential approach to improve efficacy and represents a future direction for tumor treatment^31,32^.

It is widely believed that combining ICIs with repurposed drugs presents a faster and more cost-effective approach to address the limitations of ICIs in tumor immunotherapy, especially in patients with advanced malignant tumors and multidrug resistance.

## Repurposing drugs for immunotherapy with ICIs (Figure 1)

**Figure 1:**
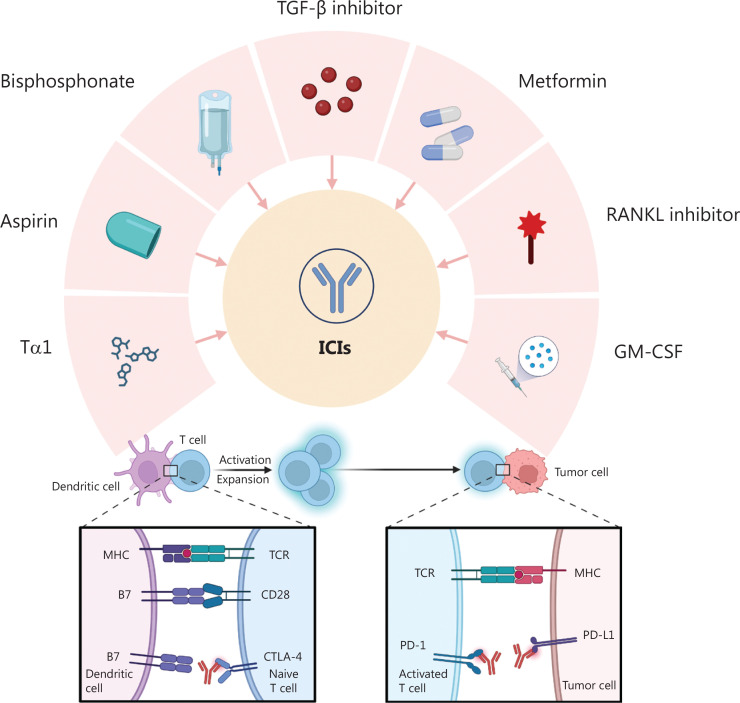
Repurposing drugs for combination therapy with ICIs. Multiple repurposed drugs, including TGF-β inhibitor, metformin, RANKL inhibitor, GM-CSF, Tα1, aspirin, and bisphosphonates, interact with immune checkpoints, such as CTLA-4 and PD-1/PD-L1. ICI, immune checkpoint inhibitor; CTLA-4, cytotoxic T lymphocyte-associated antigen-4; PD-1, programmed death 1; PD-L1, programmed cell death ligand 1; TGF-β, transforming growth factor-beta; GM-CSF, granulocyte macrophage colony-stimulating factor; RANKL, receptor activator of nuclear factor-κB ligand; Tα1, thymosin α1.

### Transforming growth factor-beta (TGF-β) inhibitor

TGF-β is a key regulator of tissue fibrosis, the overactivity of which is associated with idiopathic pulmonary fibrosis, systemic sclerosis, and liver fibrosis. TGF-β inhibitors were initially developed as potential treatments to mitigate fibrotic tissue remodeling and scarring. During tumor progression, TGF-β is secreted by tumor cells, mesenchymal fibroblasts, and other cells within the TME. TGF-β not only enhances tumor structural complexity, which supports rapid growth, but also suppresses the anti-tumor immune response by inhibiting immune cell activity, ultimately facilitating tumor cell escape from immune surveillance^33^. Inhibition of immune cell activity is a critical mechanism in the progression of malignant tumors^34^. TGF-β inhibits the activation and proliferation of immune cells by reducing IL-2 secretion and hindering the maturation and infiltration of DCs, which are crucial for antigen presentation. Moreover, TGF-β skews CD4+ T cells to Tregs and shifts macrophages and neutrophils to anti-inflammatory phenotypes, which promote tumor growth and metastasis *via* various mechanisms, such as angiogenesis, lymphangiogenesis, and epithelial-mesenchymal transition (EMT). Conversely, pro-inflammatory macrophages and neutrophils have a positive role in anti-tumor immunity by releasing cytokines and proteases that support immune activation and tissue remodeling^35,36^.

Dodagatta-Marri et al. investigated the effects of anti-PD-1, anti-TGF-β, and combination therapies on squamous cell carcinoma (SCC) implanted into syngeneic FVB mice. The study revealed a TGF-β-mediated immunosuppressive response exhibited by SSC following anti-PD-1 treatment, which led to reduced effectiveness of anti-PD-1 therapy alone. The results demonstrated that anti-PD-1 monotherapy skewed the CD4+ Treg:CD4+ Th ratio and promoted tumor cell pSmad3 expression, which was reversed upon treatment with α-TGF-β antibodies^37^. Another study tested a combination treatment approach using SRK-181-mIgG1, an antibody that blocks the activation of latent TGFβ-1, and an anti-PD-1 antibody. The study was conducted using the MBT-2 bladder cancer, the Cloudman S91 melanoma, and the EMT-6 breast cancer mouse models, which did not respond to anti-PD-1 treatment alone. Combination therapy induced a significant anti-tumor response and increased the survival rate, which was associated with an increase in CD8+ T cells within the TME and a decrease in immunosuppressive myeloid cells^38^. Additionally, Mariathasan et al. developed mouse models of EMT-6 and MC38 that mimicked the excluded tumor-immune phenotype and demonstrated that the combined use of TGF-β-blocking and anti-PD-L1 antibodies reduced TGF-β signaling in stromal cells. This model allows T cells to penetrate tumor centers, which in turn stimulates a robust anti-tumor immune response and results in tumor regression^39^. Similar effects were validated by Qin et al. using pirfenidone, a TGF-β signaling suppressor, together with PD-L1 blockade in mouse models of lung cancer, colorectal adenocarcinoma, and hepatocellular carcinoma^40^. Panagi et al. investigated the repurposing of the TGF-β inhibitor, tranilast, a drug approved for anti-fibrotic and anti-histamine treatment in the triple-negative breast cancer (TNBC) microenvironment. The authors used a combination immunotherapy cocktail consisting of PD-1 and CTLA-4 antibodies, Doxil nanomedicine, and tranilast to evaluate the efficacy on tumor growth. The results revealed that neither the immunotherapy cocktail nor Doxil monotherapy affected tumor growth, whereas a combination of the three components (PD-1 and CTLA-4 antibodies, Doxil nanomedicine, and tranilast) resulted in a significant reduction exceeding 50% compared to the untreated group. Flow cytometry analysis of the T cell populations in these tumors revealed that the triple combination significantly reduced intra-tumoral Foxp3+ Tregs and yielded a 7-fold increase in the Foxp3+:Treg ratio in the cytotoxic CD8+ T cell population, suggesting that TGF-β inhibition combined with cytotoxic nanomedicine could be a promising therapeutic strategy for TNBC microenvironment normalization and improve anti-tumor immunity^41^. Further research has demonstrated that sequential TGF-β inhibition and PD-1 blockade, rather than simultaneous combination therapy, achieves better disease control^42^. Holmgaard et al. investigated the effects of combining galunisertib, a small molecule inhibitor of TGF-βRI, with anti-PD-L1 checkpoint blockade to augment and expedite the immune-related gene expression profile within the TME compared to treatment with anti-PD-L1 alone. Holmgaard et al. suggested that in colon carcinoma models, combination therapy yields superior immune activation outcomes^43^. Tauriello et al. demonstrated the effectiveness of galunisertib in mice with progressive metastatic liver disease. This treatment sensitized tumors to anti-PD-1/PD-L1 therapy, as evidenced by improved outcomes^44^.

In a multinational phase Ib study, Melisi et al. evaluated the combination of galunisertib with the anti-PD-L1 antibody, durvalumab, for the treatment of metastatic pancreatic cancer. Although the combination therapy was well-tolerated, the clinical response rate was limited, with a disease control rate of 25.0%. Further studies are required to identify predictive biomarkers of TGF-β inhibition and optimize therapeutic strategies^45^. Ongoing clinical trials (NCT02734160, NCT02699515, NCT03315871, NCT02423343, and NCT02947165) are investigating the potential of TGF-β targeting antibodies in combination with anti-PD-1/PD-L1 immunotherapy, and may provide promising evidence for the development of combination therapies to enhance the efficacy of existing immunotherapies for cancer treatment.

### Metformin

Metformin, a widely used drug to treat type 2 diabetes, has gained attention as a potential therapeutic option for cancer prevention and treatment^46,47^. The effects of metformin on immune cells, immune-related molecules, and the TME have been extensively studied, which has shown the impact of metformin on anti-tumor immune responses that influence tumor proliferation, metastasis, drug resistance, and immunotherapy outcomes. The mechanisms by which metformin exerts its effects are diverse, including modulation of classical 5′-adenosine monophosphate-activated protein kinase (AMPK) signaling, anti-tumor angiogenesis, targeting of tumor stem cells, and regulation of insulin-like growth factor. Recent studies have demonstrated that the intestinal flora is critical for tumor immunotherapy, and that metformin also participates in regulation of the microbiota and affects the progression and treatment of tumors^48–50^. Metformin may serve as an adjuvant for ICIs and promote immune-mediated tumor clearance by overcoming or alleviating tumor-induced immunosuppression.

Cha et al. reported that metformin treatment leads to decreased expression and membrane localization of PD-L1. Decreased expression and membrane localization of PD-L1 was shown to enhance the activity of cytotoxic T lymphocytes (CTLs) against cancer cells in a 4T1 breast tumor model. By blocking the inhibitory signal of PD-L1, metformin has the potential to improve the efficacy of immunotherapy. Furthermore, the combination of metformin and CTLA-4 blockade may further enhance this effect, suggesting a potential strategy for improving cancer immunotherapy^46^. The use of metformin to enhance CTL activation by blocking the PD-L1/PD-1 signaling pathway could represent a promising therapeutic approach in tumors characterized by elevated PD-L1 expression and significant infiltration of CTLs. Metformin may also improve the response to ICI therapy in patients with non-alcoholic steatohepatitis (NASH)-related hepatocellular carcinoma (HCC). In a study by Wabitsch et al., multiple murine NASH models were used to better understand the effect of NASH on ICI therapy in patients with HCC. The results revealed that NASH induced metabolic dysfunction in CD8+ T cells, which led to reduced motility. Treatment with metformin improved the metabolic fitness of CD8+ T cells and effectively restored the response to ICI therapy in NASH-related liver cancer^51^.

Afzal et al. retrospectively analyzed the medical records of 50 patients with non-small cell lung cancer (NSCLC) who received immunotherapy with or without metformin. The findings revealed that patients who received metformin in combination with immunotherapy had a higher objective response rate (ORR), disease control rate (DCR), median overall survival (mOS), and progression-free survival (PFS) than patients who received immunotherapy alone. These benefits were more pronounced in the subset of patients who received metformin and immunotherapy as second- or third-line therapy^52^. Moreover, among patients with metastatic malignant melanoma who received immunotherapy with or without metformin, the combined metformin group had a median OS of 46.7 months and a PFS of 19.8 months, which were longer than the OS and PFS in the control group^53^. Similar results have been reported in patients with advanced melanoma, lung cancer, and renal cell carcinoma^54^. Patients with tumors who receive immunotherapy exhibit a better curative effect after using metformin, which provides guiding significance for subsequent clinical trials. Pietras et al. retrospectively investigated the impact of metformin in patients with advanced or metastatic NSCLC in the OAK study. The results showed that the ORR of the atezolizumab combined with metformin group was significantly improved compared to the control group (25% *vs*. 13%; *P* = 0.038)^55^. Wang et al. reported no obvious differences in the OS, PFS, and ORR between the concurrent metformin use and control groups in patients with advanced melanoma. It is noteworthy that the combined metformin group exhibited a significantly lower average number of new metastatic sites than the control group (0.59 and 1.51, *P* = 0.009)^56^. Several clinical studies evaluating metformin in combination with immunotherapy are currently underway (NCT03994744, NCT03874000, NCT03048500, NCT03800602, NCT03618654, NCT03311308, NCT04114136, NCT04414540, and UMIN000028405).

### The receptor activator of nuclear factor-κB ligand (RANKL) inhibitor, denosumab

RANK and RANKL are important regulators of bone metabolism and immune function. Denosumab, a monoclonal antibody targeting RANKL, has been approved for use in postmenopausal women with osteoporosis to prevent skeletal complications due to metastatic tumors^57^. RANKL is widely expressed in immune cells of the myeloid system, including tumor-associated macrophages (TAMs), DCs, and MDSCs, and in lymphoid system cells, such as NK cells and Tregs^58^. Overexpression of RANKL by tumor cells upregulates PD-L1 and ILT3 expression on the surface of DCs, as well as the secretion of immunosuppressive cytokines, such as IL-10. Overexpression of RANKL leads to downregulation of histocompatibility complex class II (MHC-II) molecules and a shift towards an immune-tolerant phenotype in DCs. Additionally, effector T lymphocytes and NK cells express RANK. Moreover, RANK secreted by tumor cells hinders the anti-tumor activity of these cells^59–61^. RANKL has been proposed as a crucial coordinator of the interaction between bone biology and tumor immunology, highlighting the potential of denosumab in cancer therapy. Increasing preclinical and clinical evidence supports the concept that the elimination of RANKL-induced anti-tumor immunosuppression by denosumab enhances the anti-tumor immune response stimulated by ICIs. Mouse models have provided insight into the mechanism underlying the synergistic anti-cancer effect of this combination, which is currently being evaluated in the clinical setting.

Gómez-Aleza et al. investigated the impact of RANK signaling loss on immune cell infiltration in PyMT mouse tumor cells and found that RANKL inhibition in luminal-like breast cancer with loss of RANK signaling improved the response to anti-CTLA-4 and anti-PD-L1 immunotherapy. Similarly, denosumab also led to an increase in tumor-infiltrating lymphocytes (TILs), B and T lymphocytes, and CD4+ and CD8+ T cells within luminal and HER2+ breast tumors^62^. Ahern et al. conducted a study involving mouse models of subcutaneous and metastatic melanoma to investigate the effectiveness of co-targeting RANKL and CTLA-4. Ahern et al. reported that combination therapy was effective in suppressing subcutaneous tumor growth, and this effect was dependent on the presence of NK cells and the cytokine, IFNγ, but not CD8+ T cells. The anti-CTLA-4 IgG2a isotype in combination with anti-RANKL yielded the greatest efficacy. Additionally, combination treatment resulted in higher recruitment of CD8+ T cells into solid tumors and increased T-cell effector function^63^. Oi et al. found that CTLA-4-Ig potently inhibited RANKL-mediated osteoclast generation in a dose-dependent manner. RANKL treatment increased the expression of key osteoclast-related signaling proteins, including NFATc1 and Ctsk. Treatment with CTLA-4-Ig, however, led to suppression of the key upregulated osteoclastogenesis protein and mRNA levels^64^. Co-targeting RANKL and PD-1 effectively inhibits experimental RM1 prostate cancer and B16F10 melanoma metastasis to the lungs and improves the effectiveness of combined anti-CTLA-4 and anti-PD-1 therapy. Triple combination (anti-PD-1, anti-RANKL, and anti-CTLA-4 antibodies) was more effective in suppressing established tumor growth than dual therapy^65^.

According to Bakhru et al., in the presence of B16-GM-CSF, anti-RANKL and anti-CTLA-4 antibodies exhibited a cooperative effect, resulting in an increased frequency of CD4+ T cells expressing cytolytic markers in the TME. This additive effect results in improved survival of the host in response to a melanoma challenge^66^. Myoken et al. reported a patient with advanced mandibular osteonecrosis and bone metastases from NSCLC who was treated with a combination of pembrolizumab and denosumab. The patient achieved a partial response to metastatic NSCLC with combination therapy, which was resumed after complete removal of the necrotic bone and the infected flap^67^. Toda et al. examined the TME of giant cell tumors of the bone (GCTB). Toda et al. reported that PD-L1 expression is more frequent in patients who receive neoadjuvant denosumab therapy. Moreover, PD-L1 and signal-regulatory protein alpha (SIRPα) ICIs potentially benefit patients with GCTB and recurrent lesions after denosumab therapy because the presence of PD-L1 and higher SIRPα+ cell infiltration were highly associated with a shorter recurrence-free survival^68^. In a 2016 case report, denosumab and ipilimumab were administered to a patient with advanced metastatic melanoma and symptomatic bone metastases, and resulted in tumor shrinkage. Subsequent research has suggested that the anti-tumor efficacy of CTLA-4 and RANKL antibodies could be attributed to the cooperative action of T cells and NK cells^69^. Another case report described complete response to treatment with ipilimumab and denosumab for metastatic melanoma^70^. Similarly, Yoshida et al. presented a case of multiple metastatic melanomas treated with nivolumab and ipilimumab plus denosumab combination therapy^71^. Afzal et al. retrospectively analyzed the efficacy of RANKL and ICIs in the treatment of malignant melanoma in 2018. Afzal et al. found that combination therapy was associated with improved OS, PFS, and ORR compared to ICIs alone^72^. A 2017 observational study that analyzed real-world tumor responses in patients with NSCLC or advanced melanoma who received denosumab in combination with CTLA-4 or PD-1 inhibitors showed a significant association between longer exposure to concomitant therapy and ORR. In a retrospective evaluation of patients with malignant melanoma, the combination of denosumab with ICIs improved the median OS and PFS compared to ICI monotherapy^73^. The German Dermatologic Cooperative Oncology Group conducted a multi-center retrospective study to collect and analyze the clinical data of these patients. The study reported that the combination of nivolumab, ipilimumab, and denosumab resulted in slightly higher response rates (54%) than anti-PD-1 monoclonal antibodies plus denosumab (50%)^74^. Another retrospective observational study evaluated the efficacy of anti-RANKL agents and ICIs in patients with stage IV NSCLC and skeletal metastases. This study, including 69 patients who received denosumab within 30 days of ICI therapy, suggested that an overlap in the duration of ICI and denosumab treatment for > 3 months is associated with improved OS and PFS in patients undergoing concomitant therapy^75^. Additionally, a study presented at the 22nd World Lung Cancer Congress in 2021 explored the efficacy of bone-targeted agents (BTAs) combined with ICIs in patients with advanced NSCLC and bone metastases. The study revealed that BTA treatment was associated with improved OS and PFS in a subgroup of patients with a high bone tumor burden (HBTB). Specifically, denosumab significantly prolonged the median OS and PFS in patients with HBTB NSCLC compared to zoledronic acid, suggesting a potential synergy between ICIs and RANKL inhibitors^76^. Finally, the POPCORN study is an ongoing open-label, multi-center phase 1B/2 study involving patients with resectable NSCLC that is evaluating the efficacy and safety of combining denosumab and nivolumab preoperatively compared with nivolumab alone. The study will assess various pharmacodynamic correlations in the tumor and blood^77^. Other ongoing clinical studies include NCT03669523, NCT03161756, NCT03620019, and NCT03280667.

### Granulocyte macrophage colony-stimulating factor (GM-CSF)

GM-CSF is a multifunctional hematopoietic growth factor that promotes the differentiation of hematopoietic progenitor cells. GM-CSF is widely used to treat bone marrow suppression and leukopenia associated with bone marrow transplantation, aplastic anemia, and myelodysplastic syndrome. GM-CSF also acts as an immunostimulatory factor that enhances the differentiation, maturation, and proliferation of DCs and macrophages^78^. Activation of protein 5 and nuclear transcription factor-κB pathways *via* signal transduction and transcription is triggered by GM-CSF to promote the differentiation and maturation of DCs. This process also upregulates the expression of co-stimulatory molecules, such as MHC-II and CD80/CD86, and activates monocyte DCs. The effect of GM-CSF on granulocyte proliferation is preferential at low doses, whereas higher doses exert a stronger effect on monocyte DCs. Radiotherapy or chemotherapy directly kills tumor cells, which in turn release tumor-related antigens. The addition of GM-CSF amplifies the antigen presentation effect of the body, leading to the activation and enhancement of the anti-tumor immune response of T cells^79,80^. Therefore, GM-CSF restores the function of DCs in the “cold tumor” microenvironment, promotes tumor T cell infiltration, and converts the “cold tumor” microenvironment to a “hot tumor” microenvironment.

Animal studies have validated the effectiveness of GM-CSF combined with PD-L1 and CTLA-4 inhibitors, which enhances antigen presentation, transforms cold tumors into hot tumors, and improves the efficacy of ICIs^81^. A recent study investigated the efficacy of combining gemcitabine, cisplatin, a PD-L1 monoclonal antibody, and GM-CSF in animal models of bladder cancer. The study revealed that combination therapy significantly reduced the positive surgical margin rate in animal lesions (75% *vs*. 12.5%; *P* = 0.05) and prolonged the survival time without local recurrence (*P* < 0.0001) compared to PD-L1 inhibitor alone^82^.

A study conducted in 2020 highlighted the critical role of DCs in the treatment of tumors with PD-L1 inhibitors in which the number of mature DCs in tumor tissues was positively correlated with patient prognosis. Mature DCs are potential prognostic biomarkers for the treatment of tumors using ICIs. The efficacy of ICIs is often limited because of low autoimmunity in patients. GM-CSF can increase the number of mature DCs, promote T lymphocyte infiltration into the TME, and enhance the effects of ICIs^83^. A randomized phase 2 trial conducted by Hodi et al. reported increased survival in high-risk patients with melanoma when sargramostim was combined with ipilimumab. The study reported a 1-year survival rate of 68.9% in the combination group, thus providing further evidence for the efficacy of GM-CSF as an adjuvant therapy^84^. Clinical studies have also demonstrated the benefits of GM-CSF combined with ICIs in patients with unresectable stage III or IV melanoma in which GM-CSF improved the OS rate and reduced the incidence of adverse effects^85^. Additionally, a phase I trial showed that combining GM-CSF with CTLA-4 blockade led to clinically significant anti-tumor responses in patients with metastatic, castration-resistant prostate cancer^86^. In addition, a phase II clinical trial investigating the efficacy of ipilimumab in combination with GM-CSF in patients with metastatic melanoma demonstrated that patients with higher levels of CD8+ T cells and lower levels of CD4+ effector T cells expressing PD-1 during pretreatment may benefit from combination treatment^87^; this finding was validated by Cham et al^88^. Furthermore, combination therapy with PD-1/PD-L1 inhibitors and GM-CSF-modified tumor vaccines has demonstrated synergistic anti-tumor effects^89,90^.

### Thymosin α1 (Tα1)

In 1977 Goldstein et al. first described and characterized Tα1, which is an acidic peptide comprising 28 amino acids with N-terminal acetylation^91,92^. Application of Tα1 has propelled advances in the treatment of diseases, such as tumors, viruses, immune and autoimmune dysfunction, and infections. The immunomodulatory effects of Tα1 include improving T-cell activity, enhancing NK cell and DC activity, and improving the recognition ability and antigen presentation function of APCs^93,94^. Tα1 upregulates the expression of MHC molecules to monitor and clear tumor cells, enhance B cell lymphoma/leukemia-2 (Bcl-2) gene expression, reduce apoptotic gene expression and apoptosis of immune cells, and modulate cytokine and chemokine production to restore immunologic function^95–97^.

Studies have shown that in the context of ICIs, Tα1 exhibits the potential to enhance anti-tumor activity, while also improving safety and efficacy by regulating the differentiation and chemokine expression profile of DCs and inverting the CD8+: Treg ratio^98^. Tα1 has also demonstrated inhibitory effects on tumor metastasis and invasion by blocking STAT3-MMP2 signaling in NSCLC cells with high PD-L1 expression, suggesting a potential benefit of Tα1 combined with PD-1/PD-L1 ICIs for patients with PD-L1-positive NSCLC^99^. Moreover, combinations of Tα1 and anti-PD-1 antibodies led to significantly fewer metastases than did the vehicle in a mouse model of melanoma lung metastases^100^.

Danielli et al. reviewed cases of melanoma patients who received Tα1 in a phase II trial and the expanded access program by comparing the mOS of patients who received sequential anti-CTLA-4 inhibitors and Tα1 to assess combination therapy in long-term survivors. The study reported a significantly longer mOS (57.8 months) in the combination group compared with those patients who did not receive Tα1 (7.4 months)^101^. These findings provide a basis for further prospective studies to elucidate the immunomodulatory effects of Tα1 in combination with ICIs, particularly regarding long-term immune system changes.

### Aspirin

Aspirin, also known as acetylsalicylic acid, was initially developed and commonly used as an analgesic. Indeed, the role of aspirin as an irreversible inhibitor of cyclooxygenase (COX) enzymes, which are responsible for producing precursors involved in the synthesis of prostaglandin and thromboxane, has led to the use of aspirin in various medical applications beyond the initial use as a pain reliever and fever reducer. The immune response to diseased cells can be impaired by prostaglandin E2 (PGE2), which enables tumor cells to evade the immune system and grow rapidly^102^. COX inhibitors, such as aspirin, can impede PGE2 synthesis, thereby restoring immune system activity. Combining COX inhibitors with immunotherapy significantly reduces the progression of colorectal cancer and melanoma in mice compared to immunotherapy alone. COX inhibition, even at a modest level, improves the effectiveness of immunotherapies, including ICIs^103,104^. In a retrospective study conducted by Zelenay et al. the interaction between COX inhibitors and ICIs in patients with metastatic melanoma and NSCLC showed that combining aspirin with anti-PD-1 blockade results in rapid tumor regression and eradication of BrafV600E melanoma cells. The use of COX inhibitors during the first course of ICI treatment was associated with longer time-to-progression and ORR, but not with OS in patients with metastatic melanoma. These findings suggest that combining COX inhibitors with ICI treatment may enhance the efficacy of cancer immunotherapy and could have a significant impact on patient outcomes^105^.

### Bisphosphonates

Bisphosphonates have emerged as a promising therapeutic option for inhibiting bone resorption owing to the high affinity for bone minerals and their inhibitory effects on osteoclasts^106^. The first-generation bisphosphonates, such as chlorophosphonates, are mainly non-nitrogen-containing compounds. In contrast, the second- and third-generation drugs, which are nitrogen-containing bisphosphonates, include pamidronate (second generation) and zoledronic acid (third generation). These agents are known to interfere with the mevalonate pathway, inhibit the acryloylation of small GTPase signaling proteins, and ultimately lead to the loss of osteoclast bone destruction ability. Non-nitrogen-containing bisphosphonates, such as chlorophosphonates, accumulate cytotoxic non-hydrolytic ATP to damage osteoclasts, which also have a role in bone protection^107,108^.

Recently, numerous preclinical studies have suggested that bisphosphonates, especially zoledronic acid, exert immunoregulatory effects on the TME, thus emphasizing their potential in the treatment of malignant tumors^109,110^. Chen et al. reported that zoledronic acid, an inhibitor of macrophages, reduced PD-L1+ TAM infiltration and alleviated CD8+ T-cell suppression when combined with anti-PD-L1 therapy. This resulted in tumor growth inhibition in a mouse model of HCC^111^. Therefore, future clinical studies should explore the role of bisphosphonates in immune regulation.

## Prospect

Despite the effectiveness of ICIs in triggering lasting anti-tumor responses in a growing number of cancer types and patients, immune-refractory tumors remain a challenge. In addition to the various combinations of ICIs with other ICIs, targeted therapy, chemotherapy, and radiotherapy, there has been a focus on identifying new agents to combine with ICIs, particularly through drug repurposing. In addition to the several drugs mentioned in this article, other drugs, such as steroids, statins, angiotensin receptor blockers (ARBs), antidepressant drugs, selective serotonin reuptake inhibitors (SSRIs), propranolol, and vitamin D, are being studied for their roles in improving the ICI response. Repurposing of these drugs in combination with ICIs is an exciting area of research in oncology. Repurposing offers the possibility of improving treatment outcomes, reducing side effects, and expanding the range of cancers that can benefit from immunotherapy. Clinical studies are ongoing to further validate the safety and effectiveness of these combinations. Thus, we suggest further research in this area, including conducting more clinical trials and the accumulation of real-world clinical data regarding the combination of repurposed drugs and immunotherapy.
